# The regulation of angiogenesis by tissue cell-macrophage interactions

**DOI:** 10.3389/fphys.2014.00262

**Published:** 2014-07-09

**Authors:** Michal A. Rahat, Bernhard Hemmerlein, Vijaya Iragavarapu-Charyulu

**Affiliations:** ^1^Immunology Research Unit, Carmel Medical Center and The Ruth and Bruce Rappaport Faculty of Medicine, Technion-Israel Institute for TechnlogyHaifa, Israel; ^2^Department of Pathology, Georg-August University HospitalGöttingen, Germany; ^3^HELIOS-Klinikum, Institut für PathologieKrefeld, Germany; ^4^Tumor Immunology, Department of Biomedical Sciences, Florida Atlantic UniversityBoca Raton, FL, USA

**Keywords:** tumor cells, chemokines, semaphorins, chitinases, EMMPRIN, nitric oxide, hypoxia, radiation

Angiogenesis, the sprouting of new blood vessels from existing ones, is a process important both in physiological and pathogenic conditions. During angiogenesis, endothelial cells proliferate, migrate and organize into new, functional blood vessels. Macrophages play an important role in this process as they process microenvironmental cues, and directly secrete or stimulate other cell types to secrete pro-angiogenic mediators, including chemokines, cytokines and growth factors that together stimulate endothelial cell proliferation, degrade the extracellular matrix (ECM), and attract leukocytes to further enhance angiogenesis. Since macrophages are extremely plastic and can be differently activated by various stimuli, they exert many regulatory effects on angiogenesis, although many aspects of this regulation are still unclear.

This volume highlights several examples of macrophages-driven regulation of angiogenesis. We begin with an introductory review which describes the role of tumor-associated macrophages (TAMs) in angiogenesis and lymphangiogenesis. Riabov et al. (2014) describe the crosstalk between tumor cells and TAMs in hypoxic and cytokine-rich microenvironment, resulting in the induction of pro-angiogenic behavior in both cell types.

We then chose to highlight the role of the chitinases, a family of proteins with newly discovered roles in angiogenesis. In this review by Shao (2013), the roles and mode of action of YKL-40/chitinase-3-like-1 (CHI3L1) are described focusing on its angiogenic signature pertaining to tumor vascularization and development. YKL-40 regulates tumor vascularization mediated by endothelial cells and promotes vascular integrity supported by smooth muscle cells. Shao also reports that while YKL-40-induced angiogenic response in endothelial cell is VEGF-independent, YKL-40 itself is induced when VEGF is inhibited.

Next, an original paper by Libreros et al. (2013) suggests that increased levels of CHI3L1 produced by pulmonary macrophages set up the pre-metastatic niche in breast cancer. CHI3L1 enhances angiogenesis by inducing chemokines (CCL2 and CXCL2) and MMP-9 expression, and attraction of macrophages into the lung. The importance of CHI3L1 as a regulator of angiogenesis, as well as possible target of treatment, is demonstrated by the addition of chitin microparticles that can inhibit CHI3L1, chemokines and MMP-9 production in the pre-metastatic lung.

Several pro-inflammatory mediators are long known to be involved in angiogenesis, and the next three reviews elaborate on these. Owen and Mohamadzadeh (2013) describe the potential roles of M2 TAMs in modulating angiogenesis through production of chemokines. The role of the pro-angiogenic ELR+ chemokines that attract neutrophils, and the angiostatic ELR-chemokines, with the exception of CXCL12, are discussed in the context of tumor progression. M1 vs. M2 secreted profiles of cytokines/chemokines which may play roles in immune regulation, angiogenesis, tumor progression and metastasis are highlighted.

Voronov et al. (2014) describe the roles of the IL-1 family of proteins in angiogenesis, and especially IL-1β that can directly drive endothelial cells to proliferate and generate tube-like structures, and to secrete pro-angiogenic cytokines and chemokines. Additionally, IL-1α attracts macrophages and collaborates with VEGF to enhance angiogenesis. Knockout mice for each of the different members of the family help reveal that IL-1β, and to a lesser extent IL-1α, are responsible for the pro-angiogenic effects, and therefore IL-1β depletion could reduce angiogenesis.

The dual role of nitric oxide in tumor biology, as a cytotoxic factor for tumor cells, and as a pro-angiogenic factor, is summarized by Rahat and Hemmerlein (2013). The authors caution against the common use of iNOS immunohistochemical staining as a prognostic factor, as no correlation between survival rates, invasiveness or tumor recurrence after therapy is found. This is due to the ability of the microenvironment (e.g. hypoxia) to inhibit iNOS activity, even when it is highly expressed. Furthermore, tumor cells can manipulate their own expression of iNOS through the expression of microRNA-146a, to control their fate and evade macrophage-induced cell death, suggesting new possible therapeutic approaches.

Two original papers demonstrate the pro-angiogenic role of additional, less studied proteins. Garcia-Areas et al. (2014) suggest that semphorin 7A (SEMA7A), a member of the semaphorin family involved in neuronal axonal guidance, is also a pro-angiogenic factor whose expression is increased in tumor-bearing mice. When triggered by SEMA7A, macrophages elevate secretion of angiogenic CXCL2/MIP-2 while silencing SEMA7A resulted in decreased tumor angiogenesis, and lower levels of CXCL2/MIP-2, CXCL1 and MMP-9.

Amit-Cohen et al. (2013) report that EMMPRIN, a transmembranal protein that is overexpressed in tumor cells and have a pro-angiogenic activity, can induce the expression of MMP-9 and VEGF from macrophages in a secreted form instead of membranal form. Tumor cells and macrophages must be co-cultured for this effect, emphasizing the importance of their interaction. Secreted EMMPRIN levels were elevated through shedding-off of the membranal protein by the activity of a serine protease that was not yet identified.

The final paper sheds light on the clinical aspect of vasculature re-growth after radiation therapy. Russell and Brown (2013) argue that because radiation destroys endothelial cells and tumor vasculature, tumor hypoxia is markedly increased resulting in induction of HIF-1 and HIF-2. These factors enhance the secretion of pro-angiogenic cytokines, chemokines and growth factors and recruit bone marrow-derived monocytes and macrophages which differentiate into TAMs and TEMs in the tumor. Thus, targeting the infiltration process after radiation therapy may improve the efficiency of treatment, prevent re-establishment of new vasculature and reduce tumor recurrence.

Collectively, the articles in this topic highlight the importance and complexity of the interactions between macrophages and tumor cells in angiogenesis. Macrophages emerge as key regulators of the process because of their ability to communicate with tumor or stromal cells, sense the microenvironment and respond by inducing secretion of potent pro-angiogenic mediators. Some of these pro-angiogenic mediators and the triggers for their enhanced expression and secretion are at the focus of the current topic. Although more research is required, it is clear that tumor cell-macrophage interactions must precede and regulate the secretion phase—a point that was emphasized throughout this volume. This regulatory ability of the macrophages, and the fact that many mediators are both pro-inflammatory and pro-angiogenic, strongly links inflammation and angiogenesis together. Many inflammatory diseases, including cancer, are characterized by enhanced angiogenesis and a prominent macrophage component. The findings described here may be relevant for understanding macrophage-angiogenesis networks and potential therapy designs.

## Conflict of interest statement

The authors declare that the research was conducted in the absence of any commercial or financial relationships that could be construed as a potential conflict of interest.
